# Letter to the editor: clarifying some aspects and the terminology of individualized human milk fortification

**DOI:** 10.1186/s12887-019-1491-x

**Published:** 2019-04-26

**Authors:** Sertac Arslanoglu, Caroline King, Clair-Yves Boquien, Delphine Lamireau, Paola Tonetto, Barbara Krolak-Olejnik, Jean-Charles Picaud

**Affiliations:** 1 European Milk Bank Association (EMBA) Working Group on Human Milk Fortification, Milan, Italy; 2 Division of Neonatology, Department of Pediatrics, Istanbul Medeniyet University, Istanbul, Turkey; 3 Department of Nutrition & Dietetics, Imperial College Healthcare NHS Trust, London, UK; 4 PhAN, Institut National de la Recherche Agronomique (INRA), Université de Nantes, CRNH-Ouest, Nantes, France; 5 Lactariums de Bordeaux-Marmande, Pôle pédiatrique, Centre Hospitalo-universitaire (CHU) de Bordeaux, Bordeaux, France; 6 City of Health and Science of Turin, Neonatal Unit of Turin University, Turin, Italy; 7 Division of Neonatology, Wroclaw Medical University, Wroclaw, Poland; 8 Division of Neonatology, Hôpital de la Croix-Rousse, Lyon, France; 9 Laboratoire CarMeN, INSERM U1060, INRA U1397, Université Claude Bernard Lyon 1, Lyon, France

**Keywords:** Human milk fortification, Adjustable fortification, Individualized fortification, Preterm infants, Targeted fortification, Preterm infant feeding, Enteral nutrition, Blood urea nitrogen

## Abstract

This letter has been written by the components of the European Milk Bank Association (EMBA) Working Group on Human Milk Fortification in response to a recent paper published by Mathes et al. (BMC Pediatr. 2018 May 8;18(1):154) with the aim of drawing attention to the importance of the use of a metabolic marker to adapt protein intake in preterm infants. EMBA Working Group on Human Milk Fortification clarifies further the terminology and some specific aspects regarding individualized human milk fortification. There are two types of individualized human milk fortification: Adjustable human milk fortification and Targeted human milk fortification. Advantages and disadvantages of these methods are summarized.

Dear Editor,

We read with great interest the paper from Mathes et al. [1] which underlines the importance of monitoring plasma and urinary urea to adapt enteral protein intake in preterm infants. The authors aimed to obtain a practical non-invasively measured metabolic marker reflecting the short term protein intake of preterm infants. They showed that higher-protein group infants had higher plasma and urinary urea concentrations compared to lower-protein group. It is noteworthy that the authors demonstrated a highly positive correlation between plasma urea concentrations and the urinary urea-creatinine-ratio, and between actual protein intakes and plasma urea concentrations and the urinary urea-creatinine-ratio. They concluded that urinary urea to creatinine ratio might help to estimate actual protein intake in these well thriving infants.

We appreciate the attempt of Mathes et al. [1] to search for a non-invasive metabolic marker on which individualization of human milk (HM) fortification could be based. Methods employed to individualize fortification of milk fed to preterm infants should continue and adjusting protein fortification on the basis of urinary urea-creatinine ratio warrants further investigation in relation with other outcomes such as growth.

On the other hand we would like to remind them that there is a type of individualized HM fortification method, namely “adjustable fortification,” proposed in 2006 and comprises twice weekly assessments of blood urea nitrogen (BUN) as a marker of protein intake [2]. This method has been shown to be effective in improving protein intake and postnatal growth (weight gain and head circumference) in VLBW infants in the original randomized controlled trial [2] and the results have been replicated by the following observational studies [3, 4].

We are also aware that there is some confusion regarding the terminology around individualized human milk fortification, as we noticed previously [5, 6]. Therefore we are taking the opportunity to clarify this. As clearly defined in 2010 [7], there are two types of individualized fortification (Table 1): 1) Adjustable Fortification-based on regular BUN assessments; 2) Targeted Fortification- based on the macronutrient analysis of human milk.

**Table 1:** Individualized Human Milk Fortification Methods [7]

HM Fortification Method	Characteristics	Advantages/Disadvantages
1. Adjustable (ADJ) HM Fortification	BUN is monitorized twice weekly, cut-off levels of BUN are 10–16 mg/dl. If the level is less than 10 mg/dl extra protein is added to the standard fortification.	Practical, not labor intensive Monitors protein status of each infant Safeguards also against excessive protein intake Does not need expensive devices Proven to be effective in optimizing growth and protein intake with a RCT. A real individualization method taking into consideration each infant’s protein requirement
2. Targeted HM Fortification	Macronutrient concentrations in HM are analyzed and based on the results milk is supplemented with extra protein and/or fat.	Both protein and energy can be supplemented Bedside analyzers are available but are expensive May be labor intensive More importantly the method supplements the milk according to the general recommendations, does not take into consideration that each individual infant’s requirement may be different

The nutrient and energy requirements stated in the international recommendations refer to the populations not individuals. We know that some infants will require more than the recommended intakes and some less. To find out how much protein an individual infant requires it is important to monitor the physiological response of each baby to the amount received and respond accordingly. In addition, protein and energy requirements may be particularly high in subgroups of infants for example those with bronchopulmonary dysplasia or extra-uterine growth restriction. Therefore fortification of HM should be adapted to specific nutrient needs of each individual infant. Adjustable human milk fortification in this sense is a good compromise

**European Milk Bank Association (EMBA) Working Group on Human Milk Fortification**

## Abbreviations

ADJ: Adjustable
BUN: Blood urea nitrogen
EMBA: European Milk Bank Association
HM: Human milk
