# Supplementary material for: Letter to the editor: clarifying some aspects and the terminology of individualized human milk fortification
Source: BMC Pediatr. 2019 Apr 26;19:135. doi: 10.1186/s12887-019-1491-x (PMC6485054; doi:10.1186/s12887-019-1491-x)
Supplement: Related texts

# Reply: Authors’ Response to Letter-to-the-Editor (Clarifying some aspects and the terminology of individualized human milk fortification)

**Authors:** Michaela Mathes, Christoph Maas, Christine Bleeker, Julia Vek, Wolfgang Bernhard, Andreas Peter, Christian F. Poets, Axel R. Franz

**Affiliations:** 10 0000 0001 0196 8249 grid.411544.1 Department of Neonatology University Children’s Hospital, Tübingen, University Hospital, Tübingen, Germany; 11 0000 0001 0196 8249 grid.411544.1 Centre for Pediatric Clinical Studies, University Children’s Hospital, Tübingen University Hospital, Tübingen, Germany; 12 0000 0001 2190 1447 grid.10392.39 Department of Internal Medicine IV, Division of Endocrinology, Diabetology, Vascular Medicine, Nephrology and Clinical Chemistry and Pathobiochemistry, University of Tuebingen, Tübingen, Germany; 13 0000 0001 2190 1447 grid.10392.39 Institute of Diabetes Research and Metabolic Diseases (IDM) of the Helmholtz Center Munich at the University of Tuebingen, Tübingen, Germany; 14 German Center for Diabetes Research (DZD), Muenchen-Neuherberg, Tübingen, Germany

We greatly appreciate the clarification by the colleagues from the European Milk Bank Association and we agree to their comments.

According to the data we were able to present in our original article (Ref [1]), it seems that instead of measuring BUN twice weekly, measuring urinary urea or urinary urea/creatinine ratio may prove similarly effective to guide adjustable fortification of human milk in very preterm infants.
